# Marine macroalgae (*Enteromorpha intestinalis*) for improving the growth performance, meat quality traits, and serum biochemical parameters in broilers

**DOI:** 10.5455/javar.2024.k802

**Published:** 2024-06-24

**Authors:** Latifa Akter, Md. Abul Kalam, Ummay Ayman, Rafiqul Islam, Morsheda Nasrin, Sonali Bhakta, Md. Abul Hashem, Ziaul Haque

**Affiliations:** 1 Department of Anatomy and Histology, Bangladesh Agricultural University, Mymensingh, Bangladesh; 2 Department of Animal Science, Bangladesh Agricultural University, Mymensingh, Bangladesh

**Keywords:** Green macroalgae, growth performance, meat quality, serum biochemistry, seaweed

## Abstract

**Objective:**

The purpose of the current study was to examine the effectiveness of green macroalgae as a novel, natural feed additive for broilers that have a greater concentration of active ingredients.

**Materials and Methods:**

Four experimental groups of 180-day-old male broiler chicks (Cobb-500) were randomly assigned, with three replicates in each group: the control group [T0, maintained only with basal diet] and three treatment groups supplemented with macroalgae for 35 days along with basal diet [T1 = 0.05% (w/w); T2 = 0.1% (w/w); T3 = 0.2% (w/w) macroalgae]. Live weight, carcass weight, and organs’ weight were noted at the conclusion of the experiment. The meat quality was examined using the muscles of the thighs and breasts, and blood serum was obtained for biochemical assessment.

**Results:**

The results revealed that dietary supplementation of green macroalgae (0.1%) in broiler rations significantly (*p* < 0.05) improved the growth performance compared to other treated groups and controls. With increasing weight, it enhanced meat quality traits assessed by increased water holding capacity, ultimate pH, redness and yellowness, and decreased lightness of muscles in the thighs and breasts. Both the levels of serum cholesterol and abdominal fat decreased and showed no unwholesome effects on liver and kidney functions.

**Conclusion:**

For the production of safe and high-quality poultry meat, marine green macroalgae (*Enteromorpha intestinalis*) could be used as a potential feed additive. It enhanced the growth rate in broilers and improved meat quality and serum biochemical parameters for supplying healthy meat in the human food chain.

## Introduction

The economy of agriculture is greatly influenced by the production of poultry. Poultry farming is a significant agricultural subsector in several nations. The poultry sector aids the human economy by providing meat, eggs, and raw materials (feathers, waste products, etc.) for various industries, when compared to other livestock sectors. Additionally, it serves as a source of revenue and trade for individuals [1]. Due to consumers’ need for less expensive animal protein in the midst of the economic crisis, the demand for poultry meat will rise. By 2030, poultry meat consumption is estimated to increase by almost 14% globally compared to 2018–’20, where 17.8% growth is expected to be contributed by poultry meat consumption [2].

Among the most crucial elements in poultry production today are management and feeding methods (composition, systems) [3–6]. The long-standing practice of using antibiotics to treat diseases and prevent subclinical infections has certain perpetual consequences, like antibiotic-resistant pathogens emerging and residues building up in eggs as well as meat [7], which encouraged researchers to search for safe substitutes such as organic acids, probiotics, prebiotics, herbal goods, and marine natural products to improve poultry health and production efficiency [8,9]. Macroalgae/seaweeds can satisfy this demand as they are readily available, renewable biomass that is rich in biologically active components. *Enteromorpha intestinalis,* a green macroalgae, contains a higher amount of proteins, polysaccharides, polyphenols, different vitamins (i.e., Vitamin A, B1, B2, B3, B6, C, and E), and beneficial organic acids like lauric acid, behenic acid, linoleic acid, caproic acid, tridecanoic acid, myristic acid, pentadecanoic acid, tricosanoic acid, stearic acid, palmitic acid, palmitoleic acid, heptadecanoic acid, oleic acid, etc. [10,11]. The development and productivity of broiler chickens could be greatly increased by using these active ingredients as a growth enhancer [12]. Marine macroalgae have a long history of being fed to livestock as a supplement [13]. Dietary supplementation of seaweed with sheep, fish, and poultry to promote their health and immune status has been reported earlier [14–18].

The economical and effective production of chicken meat, eggs, and byproducts of high quality and safety has been dramatically improved under the modern intensive poultry production system. The poultry industry needs to prioritize healthy production and maximize production. This could be achieved by minimizing their production costs and decreasing adverse environmental impacts, where the use of macroalgae as a growth booster might be an effective alternative to the traditional antibiotic or steroid growth promoters. The use of marine macroalgae in chicken nutrition is currently gaining popularity [13,14]. When added to feed, macroalgae, which are abundant in bioactive components, can enhance the quality of the eggs and meat produced by poultry, as well as their health and production [7]. Even when supplemented at a low dietary concentration, they promote animal immunity, lipid metabolism, and gut functionality with their antiviral and antibacterial activities [19,20].

Feeding marine macroalgae is most frequently utilized as a feed supplement for hens and broilers [9,12]. The dietary value of macroalgae varies greatly and is influenced by a wide range of elements, including species, geographical origin, habitat, production region, season, harvest time, water temperature, physiological and climatic variations, etc. [21]. In light of the aforementioned discussion, we have designed the present study to find a new natural feed additive, marine green macroalgae *(E. intestinalis),* for the sake of maximizing the production of organic broilers in Bangladesh.

## Materials and Methods

### Ethical approval 

The Animal Care and Ethics Committee, AWEEC/BAU/2021(5), Bangladesh Agricultural University (BAU), Mymensingh, approved the study, which was conducted in compliance with the widely accepted guidelines for the welfare and ethics of chickens. The study was carried out in the poultry shed of the Department of Anatomy and Histology, BAU, Mymensingh-2202, from January to June 2022.

### Collection and management of birds

180-day-old male broiler chicks (Cobb-500) were used in the experiment. They were acquired from a commercial hatchery in Mymensingh, and their beginning weight was noted. Four experimental groups, each with three replicates, were created at random from the broiler chicks (per replicate, 15 chicks). For a period of 35 days, the treatment groups received an additional supplement of powdered green macroalgae, whereas the control group (T0) was fed only a basal diet [T1 = basal diet + macroalgae 0.05% (w/w); T2 = basal diet + macroalgae 0.1% (w/w); T3 = basal diet + macroalgae 0.2% (w/w)] (Table 1). Standard management procedures were followed to care for the broiler chicks, including feeding, cleaning, immunization schedules, and record keeping. The temperature in the poultry house was kept between 32°C–34°C and 23°C from the first week until the end of the experiment, with a relative humidity of about 50%–60%.

**Table 1.** Experimental diet composition.

Ingredients	%
Maize	60.53
Protein concentrate	3.31
Rice polish	5.00
Soybean meal	25.00
Limestone powder	0.91
Di-calcium phosphate (DCP)	1.68
Soybean oil	2.51
Lysine	0.23
Methionine	0.16
Coccidiostat	0.05
Vitamin mineral premix	0.15
Choline chloride	0.07
Salt	0.41
**Estimated nutrients level**	
Metabolizable energy (Kcal/Kg Dry Matter)	3050
Crude protein %	21
Calcium %	0.96
Total phosphorous %	0.75
Available phosphorous %	0.46
Lysine %	1.20
Methionine %	0.51

### Collection of samples

10 broilers from each group were randomly chosen at the conclusion of the experiment, and they were killed by cervical dislocation. Before sacrifice, the final body weight was recorded to assess the effect of green macroalgae on the growth performance of broilers. The carcass weight was also measured after removing all the feathers and visceral organs. The liver, pancreas, heart, spleen, gizzard, and bursa of Fabricius were collected for weight measurement. To gain insight into the biochemical profile of serum in broilers, blood samples were collected. Chickens were kept in a fasting condition for 12 h and blood was collected from the axillary vein using a needle and syringe. After the collection of blood, it was centrifuged at 3,000 rpm for 10 min and the serum samples were kept in Eppendorf tubes. Until analysis, the serum samples were stored at -20°C. On the other hand, breast and thigh muscles were also collected for the evaluation of the meat quality.

### Serum biochemical profiles 

All the serum samples were analyzed for liver enzymes (alanine aminotransferase, ALT, and aspartate aminotransferase, AST), total cholesterol, and creatinine. All these parameters were analyzed from the collected blood serum using an automatic analyzer 902 (Hitachi, Germany).

### Meat quality

#### Meat color

To measure the surface color of the collected broiler meat samples, a Chroma Meter (CR-400; Minolta Co., Osaka, Japan) was used. Deboned meat samples of 2–3 cm thickness were used to avoid background influence. The posterior surface of the skinless breast and thigh muscles was chosen for evaluation of the meat color. However, Commission Internationale de l’Éclairage (CIE) values were used to express the meat color, wherein the meat samples’ redness, yellowness, and lightness are indicated by a*, b*, and L*. Subsequently, the values underwent analysis to examine the variation in color of the meat samples [22].

#### Ultimate pH (pHu)

The pHu of the meat sample was measured using a pH meter. At a temperature of 24°C, the reading of the pH meter was calibrated to 7.00 using a neutral buffer solution. Each meat sample was subjected to a pH assessment in three distinct areas. After that, the mean value was calculated, and every data point was carefully recorded [22].

### Water holding capacity (WHC)

The WHC of meat samples was measured using the centrifugation method. Here, we weighed and chopped 1 g (W0) of thigh and breast meat with a meat cleaver from each sample. After the meat was chopped, it was transferred to a PCR tube, and the combined weight of the tube and sample, W1, was measured. The tubes were then centrifuged (4°C) for 10 min at 10,000 RCF (relative centrifugal force). After that, a micropipette was used to discard the supernatant fluid. W2 was recorded as the new sample-and-tube weight that had been measured [22].

The final step was to calculate the WHC by applying the formula: WHC (%) = [1 – {(W1–W2)/W0}] × 100.

### Statistical analysis

The dataset’s normality was assessed using the Shapiro-Wilk test. Data were gathered during the period of study, and statistical analysis was performed using GraphPad Prism (version 9.0) by the One-Way ANOVA technique with a post hoc Tukey’s multiple comparisons test following a completely randomized design. Multiple comparisons were used to differentiate significant means at the 5% significance level. All data points (*n*-numbers) are plotted in each bar graph (three independent experiments).

**Figure 1.**
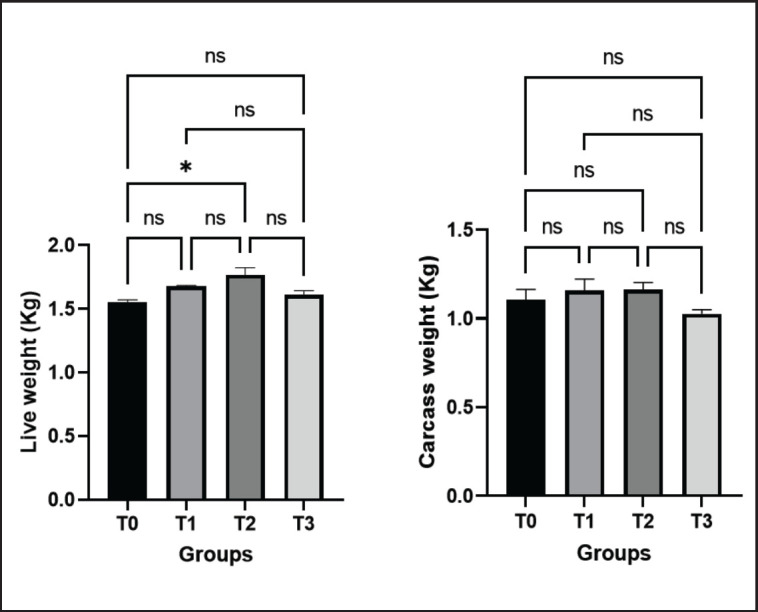
Effects of green macroalgae on live weight and carcass weight of broilers (Mean ± SEM). T0 = control, T1 = 0.05% green macroalgae, T2 = 0.1% green macroalgae, T3 = 0.2% green macroalgae with basal diet. Significance was considered at the level of 5% (*p* < 0.05). *p ≤ 0.05, **p ≤ 0.01, ***p ≤ 0.001, ns-not significant.

## Results

### Effects of green macroalgae on the live weight and carcass weight of birds

The live weights of control (T0) and treated birds (T1, T2, and T3) were 1.551 ± 0.021 kg, 1.678 ± 0.008 kg, 1.767 ± 0.058 kg, and 1.613 ± 0.031 kg, respectively. The data showed that the live weight of broilers increased in all the treated groups but significantly increased in the T2 group (*p* < 0.05) in comparison to the control group. Although the carcass weight data displayed a non-significant increase in the T1 and T2 groups, it slightly decreased in the T3 group in comparison to the control group (T0) (Fig. 1).

### Effect of green macroalgae on offals’ weight

**Figure 2.**
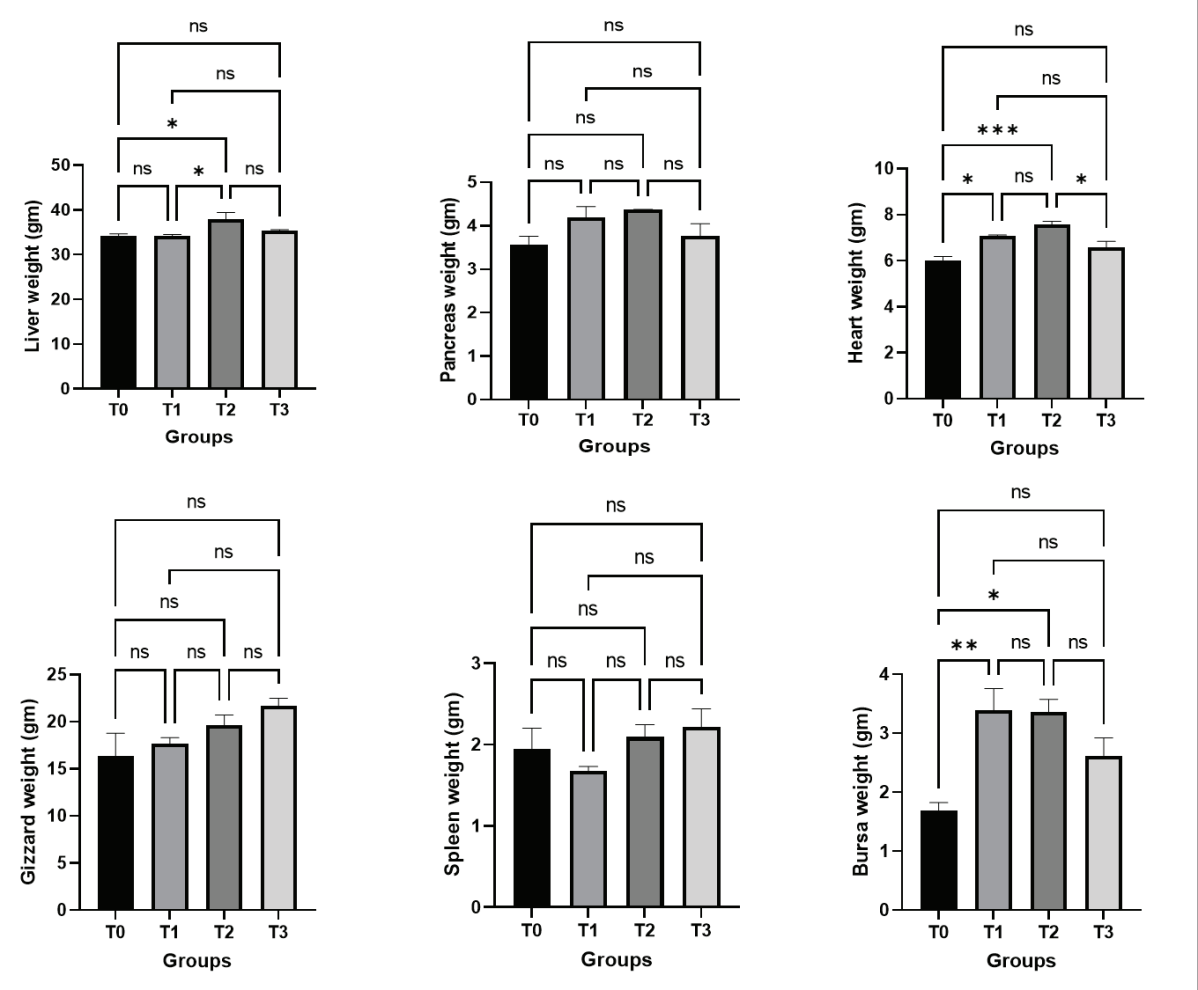
Effects of green macroalgae on different organs’ weight (Mean ± SEM). T0 = control, T1 = 0.05% green macroalgae, T2 = 0.1% green macroalgae, T3 = 0.2% green macroalgae with basal diet. Significance was considered at the level of 5% (*p* < 0.05). *p ≤ 0.05, **p ≤ 0.01, ***p ≤ 0.001, ns-not significant.

Experimental results revealed that the addition of green macroalgae gradually increased the weight of the liver, pancreas, heart, and bursa in the T1 and T3 groups but decreased in the T3 group. However, the most significant outcomes in the liver (*p* < 0.05), heart (*p* < 0.001), and bursa (*p* < 0.05) were observed in the T2 group in comparison to the control group (T0). Unexpectedly, seaweed caused a non-significant decrease in spleen weight in the treatment group T1, but gradually increased in the T2 and T3 groups (Fig. 2). In the case of gizzard, a non-significant increase in weight was found in the T3 group compared to the control group.

### Effect of green macroalgae on meat quality

#### WHC of breast and thigh meat

**Figure 3.**
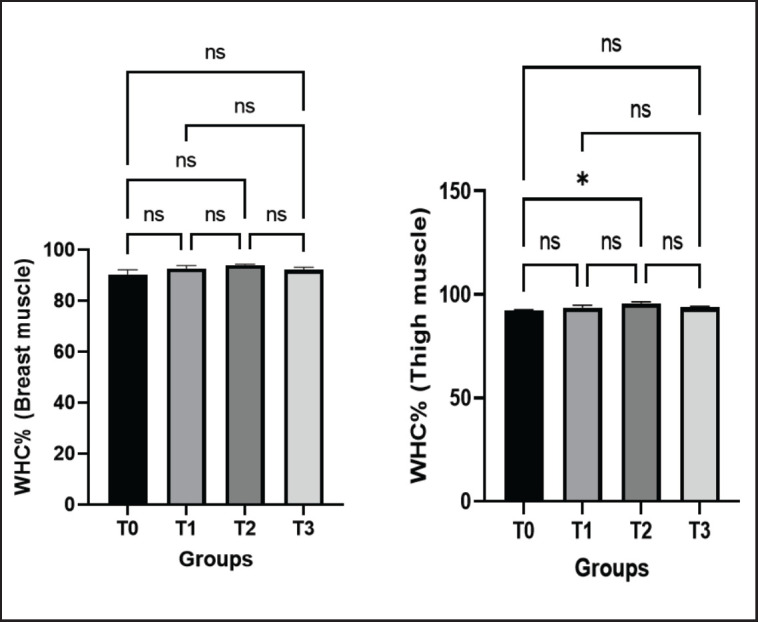
Effects of green macroalgae on WHC of breast and thigh muscle (Mean ± SEM). T0 = control, T1 = 0.05% green macroalgae, T2 = 0.1% green macroalgae, T3 = 0.2% green macroalgae with basal diet. Significance was considered at the level of 5% (*p *< 0.05). *p ≤ 0.05, **p ≤ 0.01, ***p ≤ 0.001, ns-not significant.

The WHC of breast muscle and thigh muscle in control (T0) and treated birds (T1, T2, and T3) were 84.47% ± 1.80%, 86.67% ± 1.31%, 88.13% ± 0.34%, 86.27% ± 1.06%, and 85.33% ± 0.22%, 86.67% ± 1.17%, 89.73% ± 0.72%, and 87.8% ± 0.38%, respectively. In breast muscle, a non-significant increase was noticed in all groups, whereas in the thigh muscle, a significant outcome was recorded in group T2 (*p* < 0.05) when compared to group T0 (Fig. 3). Visual appeal, weight loss, cooking yield, and sensory characteristics after consumption are all influenced by the water-holding capacity of meat products. Thus, these results indicated the positive impacts of green macroalgae on the meat quality of broilers.

#### pHu of breast and thigh meat

**Figure 4.**
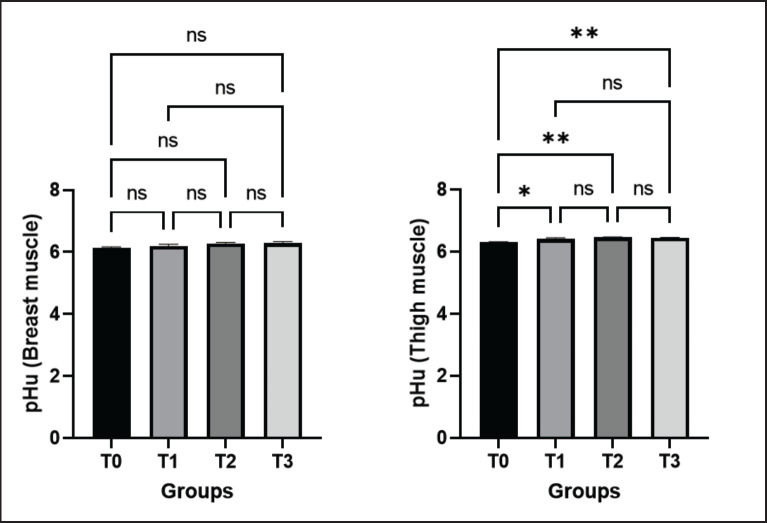
Effects of green macroalgae on pHu of breast and thigh muscle (Mean ± SEM). T0 = control, T1 = 0.05% green macroalgae, T2 = 0.1% green macroalgae, T3 = 0.2% green macroalgae with basal diet. Significance was considered at the level of 5% (*p* < 0.05). *p ≤ 0.05, **p ≤ 0.01, ***p ≤ 0.001, ns-not significant.

The pHu of the breast meat in control (T0) and treated groups (T1, T2, T3) were 6.14 ± 0.02, 6.19 ± 0.06, 6.28 ± 0.03, and 6.29 ± 0.05, respectively. A positive relationship was found between the dose rate of macroalgae and pHu in relation to the control group in the case of breast muscle. However, no significant increase in pHu was observed in breast muscle in the treated groups. An increasing tendency of pHu was also perceived in the thigh muscle, with significance at the level of *p* < 0.05 in T1 and *p* < 0.01 in T2 and T3 groups, contrary to the control group (Fig. 4).

#### Redness (a*), yellowness (b*), and lightness (L*) of breast and thigh muscles

**Figure 5.**
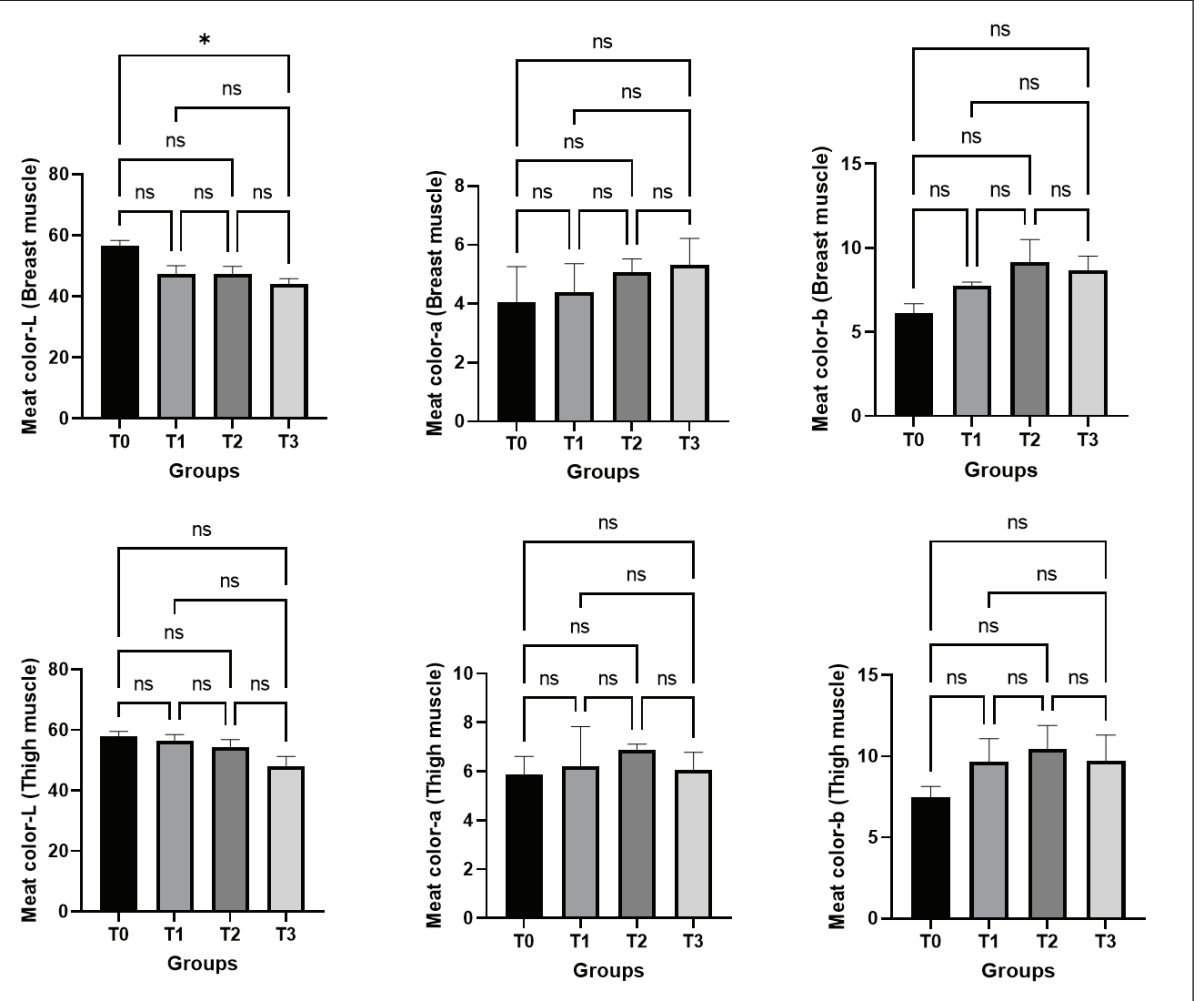
Effects of green macroalgae on lightness (L), redness (a), yellowness (b) and of breast and thigh muscle (Mean ± SEM). T0 = control, T1 = 0.05% green macroalgae, T2 = 0.1% green macroalgae, T3 = 0.2% green macroalgae with basal diet. Significance was considered at the level of 5% (*p *< 0.05). *p ≤ 0.05, **p ≤ 0.01, ***p ≤ 0.001, ns-not significant.

The redness (a*), yellowness (b*), and lightness (L*) of both the breast and thigh muscles were presented in Figure 5. The result of the current experiment revealed that in both the breast and thigh muscles, the redness in treated groups gradually increased along with the increasing concentration of macroalgae, but not significantly. However, in thigh meat, the highest value was notified in the T2 group. When considering the b* of examined muscles, an increasing pattern of value in treated groups was found on either muscle when compared to the control group (T0), with the highest value in group T2 (Fig. 5).

Decreasing the lightness of muscle is one of the indicators of improved muscle quality. According to the results, in breast meat, a non-significant decrease (47.47 ± 2.60, 47.34 ± 2.48, and 44.05 ± 1.76 in groups T1, T2, and T3, respectively) was recorded in treated groups compared to the control group (56.51 ± 1.85). However, lightness significantly (*p* < 0.05) decreased in the T3 group. In thigh meat, a non-significant decrease in lightness was also apparent, which was also represented in the analytical data. That means the addition of macroalgae in the basal diet at different concentrations resulted in a reduction in the lightness of thigh muscle (Fig. 5).

### Effect of green macroalgae on abdominal fat content (%)

**Figure 6.**
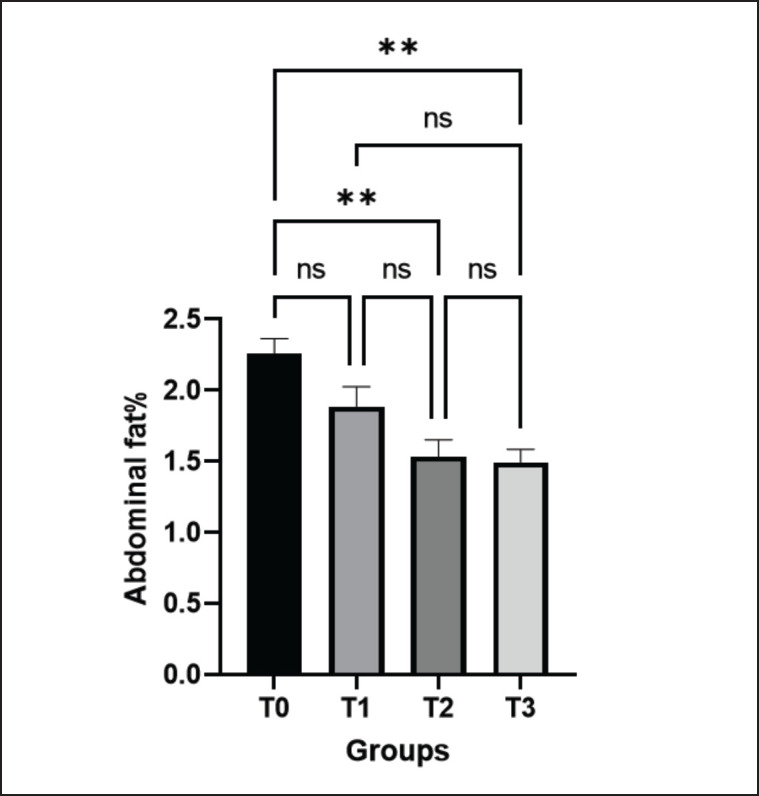
Effects of green macroalgae on abdominal fat (%) in broilers (Mean ± SEM). T0 = control, T1 = 0.05% green macroalgae, T2 = 0.1% green macroalgae, T3 = 0.2% green macroalgae. Significance was considered at the level of 5% (*p* < 0.05). *p ≤ 0.05, **p ≤ 0.01, ***p ≤ 0.001, ns-not significant.

The abdominal fat (%) of control (T0) and treated birds (T1, T2, and T3) were 2.26% ± 0.10%, 1.88% ± 0.14%, 1.54% ± 0.12%, and 1.49% ± 0.09%, respectively. The results showed that the abdominal fat of the broilers decreased in all the treated groups, but significantly (*p* < 0.01) decreased in the T2 and T3 groups in comparison to the control group (Fig. 6). The decreased abdominal fat percentage suggested safer broiler meat production for public health.

### Effect of green macroalgae on serum biochemical profiles

**Figure 7.**
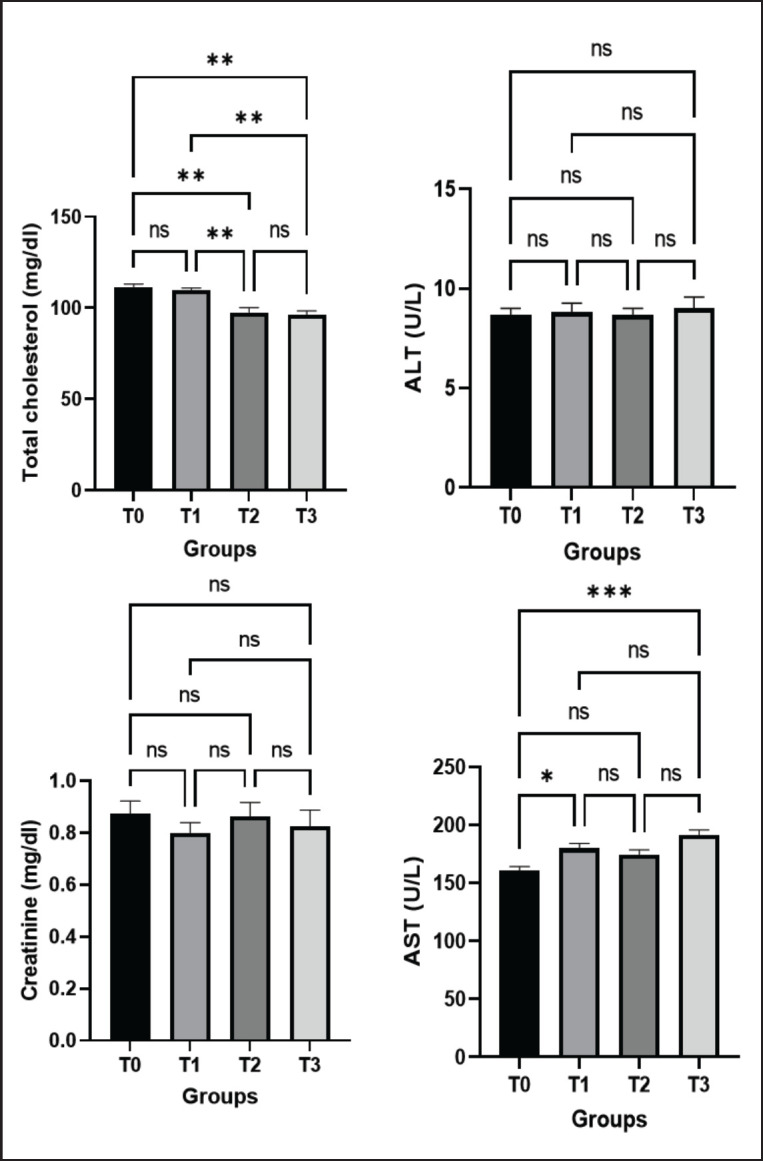
Effects of green macroalgae on serum biochemical profile in broilers (Mean ± SEM). T0 = control, T1 = 0.05% green macroalgae, T2 = 0.1% green macroalgae, T3 = 0.2% green macroalgae. Significance was considered at the level of 5% (*p* < 0.05). *p ≤ 0.05, **p ≤ 0.01, **p ≤ 0.001, ns-not significant.

Low serum cholesterol is an indicator of improved homeostasis of the blood physiology of the body. To evaluate the influence of green macroalgae in broilers, the total cholesterol of control (T0) and treated birds (T1, T2, and T3) was taken into account and recorded at 111.50 ± 1.53 mg/dl, 109.50 ± 1.29 mg/dl, 97.50 ± 2.44 mg/dl, and 96.25 ± 1.91 mg/dl, respectively. Total cholesterol levels significantly (*p* < 0.01) decreased in the T2 and T3 groups in comparison to the control group. However, the T1 group showed no noticeable changes (Fig. 7).

We also assessed the serum creatinine level of control (T0) and treated broilers and observed no significant changes among the groups (Fig. 7). The serum ALT levels of control (T0) and treated birds (T1, T2, and T3) were 8.67 ± 0.33 U/l, 8.83 ± 0.44 U/l, 8.67 ± 0.33 U/l, and 9.00 ± 0.58 U/l, respectively. The serum AST levels of control (T0) and treated birds (T1, T2, and T3) were 160.25 ± 4.33 U/l, 179.75 ± 4.91 U/l, 174.50 ± 4.58 U/l, and 191.25 ± 4.86 U/l, respectively. It significantly increased in the T1 and T3 groups compared to control birds but remained within the normal range (Fig. 7). These results suggested no remarkable effect of green macroalgae on the liver function of broilers. Together, these results declare that the supplementation of macroalgae with a basal diet in broilers does not have any unwholesome effect on the body’s physiological condition.

## Discussion

Macroalgae contain polysaccharides, which are complex carbohydrates that the upper gastrointestinal tract is unable to digest, so macroalgae are thought to be a useful source of dietary fiber [23]. The quantities of vitamins and minerals found in edible macroalgae are high enough to supplement a balanced diet [24]. In addition to this, numerous essential fatty acids are also found in macroalgae that may increase their usefulness as dietary supplements or as a component of a balanced diet [24].

### Effect of green macroalgae on body weight 

In the present study, the live weight of birds increased in all treated groups in comparison to the control group. The data on carcass weight showed an increasing weight but a slight decrease in the high-concentration group. These findings showed that broiler groups fed with green macroalgae at the proper concentration had improved carcass characteristics and increased growth performance. It could be brought on by the increased use and availability of micronutrients like protein and others, as well as the higher fiber content of macroalgae. These findings are in line with those of earlier reports where different species of macroalgae supplementation in rations demonstrated that broilers showed better or similar performance when compared to control groups [14,15,25,26]. In the present study, live weight and carcass weight slightly decreased in broilers fed with 0.2% green macroalgae, indicating higher concentrations of seaweed in the diet are hazardous. So, at proper concentration, green macroalgae can be a potential alternative to other growth boosters.

### Effect of green macroalgae on organs’ weight

The addition of macroalgae at various concentrations in the current investigation showed a favorable effect on the weight of several organs like the heart, liver, and bursa of Fabricius. In a previous study, the relative weight of the bursa of Fabricius, gizzard, and breast muscle all showed linear associations with seaweed supplementation [27]. Choi et al. [25] discovered that the addition of fermented macroalgae has a substantial impact on organ weight.

### Effect of green macroalgae on meat quality

Broiler meat quality is influenced by a number of intricate factors, and evaluating it is a challenging endeavor [28]. The water-holding capacity of meat and meat products is one of the most significant aspects of meat quality that affects cook yield, visual appeal, weight reduction, and sensory pleasures after eating [28,29]. In breast muscle, a non-significant acceleration of WHC was reported in the current study, while in thigh meat, a significant outcome was recorded in group T2. In an earlier investigation, Mir et al. [30] found that broiler breast meat with greater WHC implies increased softness and juiciness of the meat. Reduction in WHC was described by Balasubramanian et al. [27] in broilers supplemented with marine red seaweed, *Palmaria palmate. *

pH has an impact on all aspects of meat quality, including color, juiciness, tenderness, WHC, and shelf life [30]. High pH causes meat proteins to split, giving the meat a dark color. In contrast, low pH causes meat proteins to divide, giving the flesh a pale tint by allowing light to reflect off the surface unevenly [30,31]. In the current study, supplementation with green macroalgae showed a non-significant increase in pHu in treated groups in breast muscle and a significant increase in thigh muscle. As the stability of the muscle increases with an elevated level of pH, our results supported the production of more stable thigh and breast meat in broilers.

Increased redness, yellowness, and decreased lightness indicate the good quality of muscles [32,33]. The findings of the current experiment showed that, in both the thigh and breast muscles, the redness and yellowness of the breast muscle in treated groups increased as the concentration of macroalgae increased. In the current investigation, the reduction in lightness of both breast and thigh muscles was evident, indicating darker meat. The color of meat is strongly influenced by its pH, with darker meat colors being associated with higher pH levels [34]. These color coordinates showed that treated broilers’ breasts and thighs are more reddish-yellow than control broilers, illustrating improved meat quality. Similar results were described in a prior study in broilers with marine red seaweed (*Halymenia palmata*) [27].

### Effect of green macroalgae on abdominal fat (%) 

In the current study, the abdominal fat of birds decreased in all treated groups but significantly decreased in high-dosage groups. Protein and amino acids are the critical components of the broiler diet that affect abdominal fat deposition [14]. However, the decreased fat deposition might be linked to alginate compounds in the macroalgae that potentially reduce the levels of cholesterol and fat in the body [35]. Because poultry lacks alginate digestive enzymes, the alginates attach to bile salts and are eliminated through feces instead of being digested. With the increased excretion of bile salts, the liver will synthesize more of them, consuming extra cholesterol as a building block [14,35]. Reski et al. [35] also reported that broiler rations supplemented with different kinds of macroalgae may reduce abdominal fat deposition. The reduced level of abdominal fat percentage suggests safer broiler meat production for public health [36].

### Effect of green macroalgae on serum biochemical profiles

Serum biochemical profile is one of the key indicators of health status in any living being, like broilers. Low serum cholesterol is an indicator of improved homeostasis of the blood physiology of the body. In the current study, the total cholesterol levels significantly decreased in treated groups with high concentrations of macroalgae, which supports previous works with seaweed in different species [14,17,37]. Because of the addition of green macroalgae (*E. intestinalis*), this study generated lean meat that people with hypertension and cardiovascular problems may eat, as shown by the considerable drop in total cholesterol levels. 

In the current study, serum ALT and AST increased in the *Enteromorpha*-treated groups but stayed within the usual ranges. Alagan and Rajesh [37] reported in their study that the levels of ALT were within the usual range in all the groups supplemented with *U. lactuca* and *Azolla *macroalgae. The current study showed no remarkable changes in the creatinine level in treated birds. These results suggested no mentionable negative impacts of macroalgae on kidney and liver functions as well as on the body’s physiological condition in broilers.

## Conclusion

From our experimental results, we can conclude that dietary supplementation of green macroalgae (0.1%) comprehensively enhances growth performance in broilers. It also improves meat quality and serum biochemical parameters, providing healthy meat to consumers. Thus, green macroalgae* (E. intestinalis) *could be a natural, safe feed additive for quality broiler production from the consumers’ point of view.

## List of abbreviations

ALT, Alanine aminotransferase; AST, Aspartate aminotransferase; BAU, Bangladesh Agricultural University; CIE, Commission Internationale de l’Éclairage; L*, Lightness; a*, Redness; b*, Yellowness; pHu, Ultimate pH; WHC, Water holding capacity.
